# Looking beyond immediate success: unexpected challenges in the removal of novel suturing systems

**DOI:** 10.1055/a-2615-1413

**Published:** 2025-06-26

**Authors:** Elena De Cristofaro, Jérôme Rivory, Jérémie Jacques, Timothée Wallenhorst, Alexandru Lupu, Florian Rostain, Mathieu Pioche

**Affiliations:** 1 60259 Gastroenterology Unit, Department of Systems Medicine, University of Rome Tor Vergata, Rome, Italy; 2 Department of Gastroenterology and Endoscopy, Hôpital Edouard Herriot, Hospices Civils de Lyon, Lyon, France; 3 Department of Gastroenterology and Endoscopy, Dupuytren University Hospital, Limoges, France; 4 36684 Department of Gastroenterology and Endoscopy, Pontchaillou University Hospital, Rennes, France

Closure of defects after endoscopic submucosal dissection (ESD) is a routine practice to prevent complications. Conventional through-the-scope (TTS) clips are typically effective for closing linear defects up to 2 cm in size 1 . For larger defects, over-the-scope clips can be used, although their placement may hinder the use of additional closure devices 2 . To address these limitations, new suturing systems are being developed to facilitate closure after extensive ESD. Among these, the X-Tack Endoscopic HeliX Tacking System is a novel TTS device designed for soft tissue approximation and is compatible with standard gastroscopes and colonoscopes 3 .

We recently demonstrated its feasibility in closing large post-ESD defects using a combination of X-Tack and TTS clips 4 , but limited data are available on its removal, especially in patients needing mucosal surveillance.

We present the follow-up colonoscopy of a 69-year-old man with ulcerative colitis, previously treated with ESD of two adjacent lesions in the sigmoid colon (February 2024). The mucosal defect was closed using a combination of X-Tack and TTS clips ( Fig. 1 ).

**Fig. 1:**
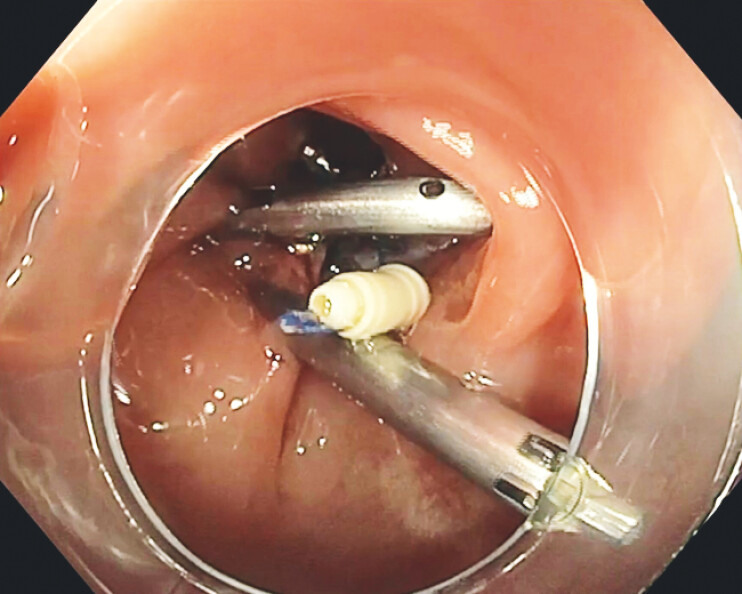
X-Tack and TTS clips placed after colonic ESD. Abbreviations: ESD, endoscopic submucosal dissection; TTS, through-the-scope.

Twelve months later, a follow-up surveillance colonoscopy revealed multiple flat subcentimetric lesions in the left colon, which were resected en bloc using a cold snare. The X-Tack system was still present, with one helix anchor embedded in the mucosa ( Fig. 2 ). To ensure complete inspection of the area, device removal was attempted. Initial removal with forceps failed. It was eventually removed using a cold snare, though with technical difficulty. A small muscularis propria injury occurred, which was successfully closed with TTS clips ( Video 1 ).

**Fig. 2:**
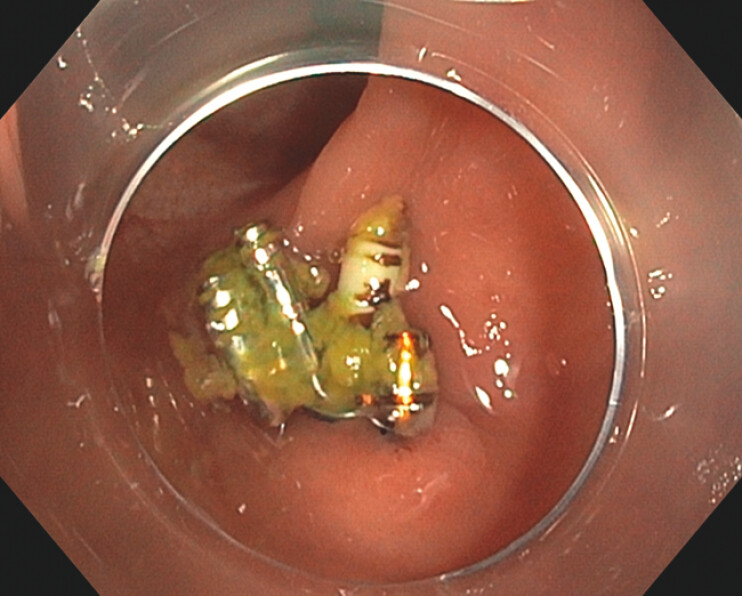
X-Tack anchor still embedded in colonic mucosa at 12-month follow-up.

Challenging removal of novel endoscopic suturing device.Video 1

This case highlights the challenges of removing anchored closure systems, especially when surveillance is required. While effective for closure, the X-Tack system may pose technical risks during removal. Careful techniques and further studies are needed to evaluate safe removal strategies across different clinical scenarios.

Endoscopy_UCTN_Code_CPL_1AJ_2AJ

## Notes

Endoscopy E-Videos https://eref.thieme.de/e-videos
*E-Videos* is an open access online section of the journal *Endoscopy* , reporting on interesting cases and new techniques in gastroenterological endoscopy. All papers include a high-quality video and are published with a Creative Commons CC-BY license. Endoscopy E-Videos qualify for HINARI discounts and waivers and eligibility is automatically checked during the submission process. We grant 100% waivers to articles whose corresponding authors are based in Group A countries and 50% waivers to those who are based in Group B countries as classified by Research4Life (see: https://www.research4life.org/access/eligibility/ ). This section has its own submission website at https://mc.manuscriptcentral.com/e-videos .
